# Conformable Fractional Deep Neural Networks (CFDNN) for high-speed cyber-attack detection

**DOI:** 10.1038/s41598-026-45213-w

**Published:** 2026-03-30

**Authors:** Basem Ajarmah, Hani Iwidat

**Affiliations:** 1 Cybersecurity, College of Administrative and Information Sciences, Al-Istiqlal University, Jericho, Palestine; 2 Data Science, College of Administrative and Information Sciences, Al-Istiqlal University, Jericho, Palestine

**Keywords:** Deep neural networks, Fractional gradient descent, Conformable fractional calculus, Cyberattack detection, NSL-KDD, Optimization, Engineering, Mathematics and computing

## Abstract

The growing sophistication of cyber-attacks exposes the limitations of conventional deep neural networks, which often suffer from slow convergence and high computational costs. This paper introduces the Conformable Fractional Deep Neural Network (CFDNN), a framework that replaces standard backpropagation with conformable fractional gradient descent. By operating in the super-integer regime ($\alpha \in [1.2, 1.8]$), the model smooths the loss landscape to accelerate training. Evaluated on NSL-KDD and CIC-IDS2018 using cross-validation, the CFDNN achieves 99.42% and 99.86% accuracy, respectively. It attains these results in just 30 epochs–a 40% reduction in training time. On the large-scale CIC-IDS2018 dataset, the model converged in approximately 24.2 minutes on a system equipped with an standard CPU. The CFDNN thus provides a computationally efficient, high-performance alternative to classical methods, offering a robust solution for modern cyber-defense.

## Introduction

The artificial neural network (ANN) is the bedrock and central component in the current evolution of artificial intelligence. Although it is truly an abstraction of the essential features of the human brain^1^, its early breadth is narrow. The principle of the deep neural network materialized in the second half of the 20th century, inspired by the need for a performance improvement from classical machine learning paradigms and the highly hierarchical human information processing architecture^2,3^.

Cyberattacks have also advanced in scale and sophistication and pose ongoing threats to governmental, financial, and industrial systems. Intrusion detection systems (IDSs), which detect malicious activity within network flows are among the most commonly used mechanisms for protection against these threats. However, traditional IDS solutions – based on signature matching and statistical rules are often disappointing in distinguishing new or zero-day attacks, thus limiting their effectiveness in rapidly dynamic environments^4,5^. Given the increasing volume and sophistication of network data, powerful models that can detect intelligent, adaptive patterns are urgently needed.

Deep learning has become a robust technique for intrusion detection, that can extract complicated, nonlinear patterns from traffic streams. These types of architectures such as convolutional neural networks (CNNs), recurrent neural networks (RNNs), and deep autoencoder, perform significantly better in terms of detection precision than classical machine learning models do^6–8^. As such the prevailing paradigm for advanced cyber-threat detection is dominated by deep learning models. While these models excel at identifying complex spatial and temporal patterns in network data, their foundation in integer-order calculus presents a fundamental constraint.

Despite such success, DNNs suffer from long-standing limitations: A critical shortcoming, as highlighted in overall assessments of deep learning, is that they do not model long-range relations and lack robust modeling of long-range relations, though fractional approaches can provide local scaling with stability benefits. That are essential for complex, multistep attacks such as advanced persistent threats (APTs)^9^. In addition, several other limitations hinder their performance in real-world applications. First, such models often require significant amounts of labeled training data; unfortunately, many intrusion detection datasets are biased or obsolete, which reduces their generalizability to new or zero-day attacks^10,11^. Second, DNNs often suffer from significant computational and training time expenses, making it difficult for them to be used in resource-constrained or real-time applications^7^. Third, problems such as vanishing gradients, overfitting and sensitivity to adversarial examples undermine their robustness and reliability in the presence of clever attack planning^12^. finaly, the vast majority of present-day^9,13^ architectures act as ”black boxes”, which offer little interpretability, hindering their use in important security applications where ability and transparency are essential^14^.

These limitations indicate that there is a need for innovative architectures that can address convergence, robustness, and adaptability enhancement with efficiency and interpretability maintenance in cyberattack detection applications. In response to such limitations, the use of fractional calculus in designing and training neural networks has emerged.

Fractional calculus is extensively employed in diffusion processes^15,16^, automation control^17–19^, image processing^20^, signal processing^21^, neural networks^22–24^ and many other domains. Owing to its long-term memory, nonlocality, and weak singularity features^22–24^, it has become a hot topic in artificial neural network research. At its heart, fractional calculus involves the differentiation and integration of arbitrary order.

Fractional-order operators extend the classical models of differentiation and integration beyond noninteger realms, thus presenting improvements in memory representation, dynamic richness, and optimization flexibility^25^. To address this limitation, fractional calculus has been proposed as a mathematically valid framework for incorporating the dynamic temporal context within neural networks. The use of fractional-order operators, which have been hailed for their distinctive nonlocal properties and memory effects, offers a principled approach to the construction of deep fractional neural networks (DFNNs). These neural systems are theoretically tailored for enhanced stealthy detection capabilities by representing not only the current points of data but also the entire past trajectory of a system’s status, thereby presenting a broader understanding of cyber threat evolution over time.

Preliminary works that utilized conformable fractional gradient descent within backpropagation have presented accelerated convergence velocities and increased stability^26,27^. Later studies have addressed fractional-order recurrent and feedforward networks, which have been able to capture deeper temporal relationships and nonlinear mappings^28^.

These developments suggest that fractional-order models transcend vast limitations that have long been associated with classical deep learning practices. The success of fractional calculus in enhancing both the performance and convergence speed of baseline gradient procedures has recently been supported within a series of works^23,29^, mostly due to kernel scaling with partial memory-like benefits, offering lower compute costs than Caputo^30^ and Riemann^31^ (20x faster in our experiments).

While both fractional-order neural networks and deep learning have offered promise singly, both combined for cybersecurity activities have not yet been sufficiently examined. Several studies have used conformable fractional neural networks in cybersecurity via various definitions, such as Caputo^30^ and Riemann^31^, which require significant computing power compared with the fractional calculus definition because of its ease of implementation and lower computational costs and speed. In particular, insufficient research has fully investigated the role of **conformable fractional deep neural networks (CFDNNs)** in enhancing intrusion detection performance on ongoing cyber-attacks.

Consequently, the integration of conformable fractional calculus with deep NNs (or MLs) has created a new paradigm of Conformable Fractional Deep Neural Networks (CFDNNs), which are theoretically better suited for capturing the complex, time-evolving characteristics of cyber-attacks^26^.

The main contributions of this study are as follows:

1. **Novel architecture:** We introduce a novel DNN architecture that incorporates conformable fractional calculus operators into deep learning layers for improved dynamics.
2. The proposed model achieves near-perfect detection performance, attaining 99.05% accuracy on NSL-KDD and 99.85% accuracy on CIC-IDS2018 with optimal fractional orders of 1.5 and 1.8 respectively. These results consistently surpass traditional methods such as logistic regression and support vector machines across all evaluation metrics.
3. This paper introduces a new way to build a strong defense for smart home systems, helping to secure them against many cyber threats.
4. **Specialized training algorithm:** We develop and evaluate a conformable fractional backpropagation algorithm for training strategies designed to accelerate convergence and enhance robustness with less computational cost.
5. **Empirical Validation and Practical Viability:** Comprehensive evaluation on benchmarks (NSL-KDD, CIC-IDS2018) shows superior detection accuracy, robustness, and efficiency for real-world cyber-defense deployment.

The research includes a detailed experimental evaluation that shows the model’s accuracy in the real-world, contributing significantly to the field of cyberattacks.

The structure of the remainder of the paper is as follows: Section “Introduction”, discusses relevant research surveys. Section “Literature review” provides details on the research methodology employed in this study. Section "Results and discussion" presents the experimental setup. Section "Background theory for conformable fractions calculus" presents the results and discussion to illustrate the effectiveness of our model. Finally, the paper is concluded, and future work is presented in section "Conclusion and Future Work".

## Literature review

This section presents the current state-of-the-art research in cybersecurity detection using conformable deep artificial neural networks, emphasizing relevant studies that apply alternative ways to leverage neural networks for cyberattack detection. The necessity for robust and adaptive cyber-attack detection systems (CADSs) has driven cybersecurity research toward advanced machine learning paradigms.

Traditional methods, particularly those based on shallow neural networks or signature matching, exhibit insufficient accuracy when confronted with the complexity of modern, remote network threats. Numerous research projects have been conducted in cybersecurity and cyberattack detection, and the topic of neural networks and deep learning remains controversial. Many researchers have attempted to build an effective and automatic system that can detect cyberattacks and intrusions via different methodologies and tools (algorithms).

This section explores existing research efforts that can assist in identifying gaps and developing the proposed model approaches.

### Evolution of intrusion detection systems (IDSs)

Different frameworks for IDSs have been used in the literature, from traditional systems such as rule-based and signature-based intrusion detection systems (IDSs) to data-driven and machine learning-based solutions, particularly those leveraging deep learning architectures^32^. Initial ML-based frameworks utilized algorithms such as k-nearest neighbors and random-forests, which were recently used for detection frameworks. These algorithms work well for finding known attack patterns but still have trouble finding new or more complicated threats.

Zhang et al.^33^ developed a multilayer data-driven system for detecting cyberattacks in industrial control systems via networks, systems, and data processing. The approach improved detection accuracy but relied heavily on labeled data and manual feature engineering.

Reference Kumar et al.^34^ developed a smart system for detecting cyberattacks on IoT networks by combining feature reduction and classification, which made it more efficient. The model faces problems with scalability and robustness. ML-based intrusion detection systems based on decision tree models and standard datasets were used in^35^. The model was constrained to shallow models that face novel attacks.

Machine learning (ML) and deep learning (DL) are powerful tools for accurate cyberattack detection^12,36,37^. Numerous studies have explored different ML architectures to improve cyper-attack detection.

In^38^ a data mining framework designed to derive rules that precisely distinguish the behavior of normal activities and intrusions was proposed.

^39^ developed an event classification scheme based on Bayesian networks, aiming to improve the aggregation of different model outputs, enabling the smooth integration of further information.

^40^ applied neural networks (NNs) and support vector machines (SVMs) in IDSs to identify useful patterns. However, the limitation of these traditional methods when increasing data and new attacks is accurate and timely detection; these methods are difficult to solve because of their high false alarm rates and missed alarm rates.

### Deep Neural Networks (DNNs) for detection

As a subdomain of machine learning, deep learning-based approaches have been additionally applied for intrusion detection because of their successful applications in different areas such as object detection^41^ and natural language processing (NLP)^42^.

Deep neural networks (DNNs) have been proven to be a great option, as they are better at finding hidden features, recognizing patterns, and feature extraction^8^.

^43^ employed DBN for feature extraction and Softmax and SVM for classification at the conclusion of the network. The detection accuracy of classes of attacks on the NSL-KDD dataset was 80–90%, whereas that of tiny classes was approximately 20%.

The author of^44^ a proposed recurrent neural network (RNN) cyclic NN for intrusion detection that is based on NSL-KDD. The accuracy, false negative (FN) rate, and false positive (FP) rate are superior to those of traditional ML methods.

Similarly^45^, used the ISCX2012 dataset and LSTM to perform time series classifier experiments by adding a sliding window to the dateset to obtain three-dimensional data. Compared with CNN-LSTM and other algorithms, LSTM outperforms other algorithms in terms of the recall rate and accuracy^46^. encoded the original network traffic of ISCX2012^47^ and DARPA1998^48^ directly via one-hot coding; subsequently, the CNN and CNN-LSTM algorithms were used to implement classification, respectively. Both experiments demonstrated the effectiveness of the CNN in intrusion detection; however, further improvement is required in fine-grained classification and in addressing unbalanced data.

While the NSL-KDD dataset remains a cornerstone for benchmarking IDS models due to its refined nature, it is increasingly criticized for its inability to represent modern, sophisticated network traffic patterns and zero-day threats. Research that focuses exclusively on this legacy data often risks overestimating model generalizability in real-world, dynamic environments. In this study, we explicitly address these concerns by validating the proposed CFDNN on both the established NSL-KDD and the more contemporary CIC-IDS2018 dataset. This dual-benchmarking approach provides a more rigorous assessment of the model’s robustness and its ability to generalize.

### Addressing DNN optimization challenges with fractional calculus

The goal of the Conformable Fractional Gradient Descent (CFGD) approach is to leverage the unique dynamics of fractional derivatives to obtain a new optimization algorithm that is optimized, faster, and simpler than the conventional method. The noninteger order (fractional order $\alpha$) introduces a **memory effect** into the gradient calculation, allowing the optimizer to encode historical gradient information more effectively, which can stabilize the training process and potentially lead to better convergence behavior.

This approach, which uses conformable fractional calculus (CFC) for ease of deployment, uses the fractional differential gradient function^26^. A principal advantage of the fractional calculus FC approach stems from its nonlocal kernel, which introduces a memory effect into the gradient calculation. This property allows the optimizer to effectively encode historical information about previous gradients, a feature that can stabilize the optimization trajectory in the highly nonconvex, high-dimensional spaces of deep learning.

Consequently, this mechanism has been empirically linked to an optimization process that results in enhanced performance, improved convergence speed, and reduced computational complexity compared with traditional integer-order methods^26^. However, Caputo^30^ and Riemann^31^ definitions require high computing power; our conformable approach offers lower costs and faster implementation (e.g., 20x speedup vs. Caputo-based models).

Compared to modern transformer-based IDS^12^ conformable fractional scaling provides efficiency gains, achieving similar accuracy (99.05% on CIC-IDS2018) with 50% less compute than attention mechanisms^49^^26,50^. Established the mathematical basis for the FGD method, showing its convergence to various optimization systems. This confirms that the convergence rates of FGD are similar to those of traditional gradient regression^51^. However, the effects of CFGD on convergence analysis have not been fully explored.

### Research gap

Finally, while deep learning (or DNNs) has advanced intrusion detection, standard models still face several challenges. Although fractional calculus has been introduced to address these limitations by incorporating memory, the specific potential of the efficient and computationally simpler conformable fractional definition remains largely untapped for cybersecurity^52,53^. A dedicated framework that combines conformable fractional operators with a deep neural architecture and a tailored training algorithm for cyber-attack detection is absent from the literature. This work bridges that gap by proposing a Conformable Fractional Deep Neural Network (CFDNN) to enhance learning dynamics, robustness, and convergence for superior intrusion detection.

The current deep learning-based IDS research landscape is very dynamic, with recent works achieving performances on state-of-the-art metrics that are remarkably high, especially on established benchmark datasets. However, a critical review of the state-of-the-art reveals that persistent and emerging limitations constitute the current research gap.

A discrepancy is evident between the reported high accuracy rates and the generalizability of these models to real-world, dynamic network environments. Specifically, the strong reliance on the legacy NSL-KDD dataset, this limits realism; we address this by validation the CIC-IDS2018 dataset, a more modern dataset. In the majority of recent high-impact studies (e.g^54–56^.,) raises significant concerns regarding the ability of these models to detect contemporary, zero-day threats. While methods incorporating sophisticated techniques such as attention mechanisms^54^, hybrid feature selection^55^, and interpretable frameworks^57^ have pushed boundaries of accuracy, their success often remains confined to the well-trodden feature space of older data. Furthermore, complex, multistep pipelines^55,58^ introduce computational overhead and hinder the practical deployment necessary for real-time applications^59^.

Table 1 synthesizes the key models, methodologies, and findings from selected recent studies, critically highlighting their inherent strengths and crucial limitations that collectively underscore the need for a novel, robust, and generalized IDS solution capable of addressing modern threat complexity with computational efficiency.

**Table 1:** Comparison of Selected Intrusion Detection Studies.

Id	Year	Model Technique	Dataset(s) Used	FS/DR	Accuracy (or main metric)	Strengths	Limitations
^55^	2025	3ConFA feature selection + 1D-CNN (hybrid deep model with ADASYN)	NSL-KDD	Correlation + Chi^2^/IG/RFE (3ConFA)	99.56% (test accuracy)	Very high DDoS detection rate; interpretable FS via IG/SHAP; handles class imbalance (ADASYN)	**Only evaluated on NSL-KDD (older data)**; complex multi-step pipeline
^57^	2025	HDMLFS framework (ResNet-based IDS with IG+SHAP FS)	NSL-KDD; CSE-CIC-IDS2018	Correlation filter + IG&SHAP voting	99.77% (NSL-KDD)/98.23% (CIC-2018)	Excellent accuracy on two benchmarks; interpretable feature importance (IG & SHAP)	Feature attribution overhead; **method tuned to specific datasets**
^54^	2024	CNN with Efficient Channel Attention (small-footprint CNN)	NSL-KDD	None (used all 43 features; normalized)	99.728% (multi-class accuracy)	Extremely high accuracy; outperforms many prior models; low-complexity attention mechanism	**Evaluated only on NSL-KDD**; potential overfitting to legacy data
^56^	2023	Custom ANN (fully-connected network) + PCA dimensionality reduction	NSL-KDD	PCA (removed 13 least-varied features)	97.5% (binary classification accuracy)	High accuracy vs. multiple ML/DL baselines; faster training with fewer features (PCA)	**Only used NSL-KDD data**; PCA may omit non-linear feature relations; **limited generalizability**
^59^	2022	Deep Neural Network (fully-connected) for real-time IDS; live traffic pipeline (C++ sniffer + REST API)	NSL-KDD	Selected 28 features (excluded content features)	81% (accuracy), 96% precision, 70% recall	**Real-time deployment** (live network capture); complete system implementation for on-the-fly detection	**Moderate detection accuracy**; lower recall (misses some attacks); used outdated dataset for modern threats
^60^	2021	5-layer Autoencoder (unsupervised anomaly detector with outlier removal)	NSL-KDD	Outlier removal; AE latent-space features	90.61% accuracy (92.26% $\hbox {F}_{{1}}$)	Optimized AE architecture yields high accuracy and $\hbox {F}_{{1}}$; removing outliers lowers false alarms	**Only tested offline on NSL-KDD**; not validated on modern network traffic
^58^	2021	CNN with 2-step preprocessing (DR + deep feature synthesis)	NSL-KDD; UNSW-NB15	Yes – hybrid DR + deep feature eng.	90.1% (binary)/81.1% (multiclass)	Consistent performance on two datasets; high attack detection (recall $\approx 90\%$)	Multiclass accuracy still modest ($\approx 81\%$); **added complexity in preprocessing pipeline**

Despite significant progress, research gaps remain in the operational adoption of fractional DNNs, explainability, and robustness to adversarial attacks. The following matrix highlights the coverage of key topics and study attributes.

**Table 2:** Summary of Selected IDS Research Attributes.

Topic/Attribute	Standard DNNs	Hybrid/Ensemble	Fractional DNNs	Explain ability	Adversarial Robustness
Benchmarking on NSL-KDD	12	7	1	1	1
Benchmarking on CICIDS	10	5	1	**GAP**	**GAP**
Real-world deployment	8	3	**GAP**	**GAP**	**GAP**

## Background theory for conformable fractions calculus

In conformable fractional calculus, values of $\alpha$ exceeding unity possess meaningful theoretical underpinnings that extend beyond mere mathematical abstraction. When $\alpha$ assumes values greater than one, the operator transitions from a fractional differentiator to what can be understood as a fractional-order accelerator^61^, capturing how dynamical systems respond to perturbations with compounded sensitivity. This is particularly relevant for phenomena exhibiting memory effects where past states exert influence not linearly but through scaling relationships. The conformable derivative at $\alpha> 1$ effectively compresses the time scale, allowing the model to capture how systems accumulate and respond to historical information in ways that integer-order operators inherently cannot represent^62^. This property makes it theoretically suitable for modeling complex systems where the rate of change depends not only on current conditions but on the entire evolutionary pathway.

The algorithm is identical to standard backpropagation for the forward pass and the error backpropagation steps, but it introduces a crucial modification in the learning rate and weight update formulas as demonstrated in our previous research^26^. The fractional gradient used in the descent is based on the chain rule property of the Conformable Fractional Derivative (CFD) of order $\alpha$. The partial Conformable Fractional Derivative of the Cost Function (*C*) with respect to a weight ($W_{ij}$) is defined as:

1 $$\begin{aligned} \frac{\partial ^\alpha C}{\partial W_{ij}} = (W_{ij})^{1-\alpha } \frac{\partial C}{\partial W_{ij}} \end{aligned}$$

where:

- $W_{ij}$: The weight being updated.
- $\alpha$: Fractional order ($\alpha$ hyperparameter).
- $\frac{\partial C}{\partial W_{ij}}$: The standard (integer-order) partial derivative of the cost function with respect to the weight, which is calculated via the normal backpropagation method.

The new update rules for the weights ($W_{ij}$) and biases ($b_{i}$) in any layer are summarized in the following table:

**Table 3:** Comparison of standard and Rules fractional gradient descent update rules.

Parameter	Standard Gradient Descent ($\alpha =1$)	Fractional Gradient Descent (CFD $\alpha$)
Weight ($W_{ij}$)	$W_{ij}^{(new)}=W_{ij}^{(old)}-\eta \frac{\partial C}{\partial W_{ij}}$	$W_{ij}^{(new)}=W_{ij}^{(old)}-\eta (W_{ij}^{(old)})^{1-\alpha } \frac{\partial C}{\partial W_{ij}}$
Bias ($b_{i}$)	$b_{i}^{(new)}=b_{i}^{(old)}-\eta \frac{\partial C}{\partial b_{i}}$	$b_{i}^{(new)}=b_{i}^{(old)}-\eta (b_{i}^{(old)})^{1-\alpha } \frac{\partial C}{\partial b_{i}}$

where:

- $$\eta$$: Learning rate.
- $W_{ij}^{(old)}$ and $b_{i}^{(old)}$: The current values of the weight and bias, respectively.
- $(W_{ij}^{(old)})^{1-\alpha }$ and $(b_{i}^{(old)})^{1-\alpha }$: The scaling factor introduced by the Conformable Fractional Calculus.

### Algorithm description and implementation

The numerical implementation of the network training utilizes a modified Stochastic Gradient Descent (SGD) approach, incorporating the Conformable Fractional Derivative (CFD) to govern the parameter update laws 3. The complete logic of the training loop, including the integration of fractional order $\alpha$, mini-batch processing, and the backpropagation chain rule, is detailed in the Pseudocode: Neural Network Training (Fractional CFD Variant) provided in Appendix A. The following steps are performed:

1. **Initialization:** Initialize the network weights (*W*) and biases (*b*) with small, random values.
2. **Forward Pass:** Compute the output ($a_L$) by propagating the input forward through the network.
3. **Calculation Loss:** Compute the cost (*C*) on the basis of output and the true label.
4. **Backward Pass (Standard):** Calculate the standard (integer-order) partial derivatives $\frac{\partial C}{\partial W_{ij}}$ and $\frac{\partial C}{\partial b_{i}}$ for all weights and biases via the classical Backpropagation technique (chain rule for the error signal $\delta$).
5. **Fractional weight update (FGD):** Update the weights and biases via the fractional gradient descent equations from Table 3, incorporating $W^{1-\alpha }$ and $b^{1-\alpha }$ scaling factors.
6. **Repeat** steps 2–5 until the stopping criteria are met.

### Experimental methodology

Two benchmark datasets were employed: NSL-KDD (125,972 samples, 43 features) and CIC-IDS2018 (1,695,098 samples, 78 features). Both datasets underwent identical preprocessing: removal of constant features, elimination of perfect separators to prevent data leakage, and standardization to zero mean and unit variance. For NSL-KDD, 37 features were retained; for CIC-IDS2018, 68 features remained after preprocessing.

A deep neural network with six layers was designed: input layer matching feature dimensions, four hidden layers with descending neuron counts (32, 16, 8, 4), and a single-neuron output layer. Hidden layers utilized ReLU activation functions ($f(x) = \max (0, x)$) for non-linear transformation, while the output layer employed sigmoid activation ($\sigma (x) = 1/(1+e^{-x})$) for probability estimation. This architecture was intentionally kept consistent across experiments to isolate the effect of fractional order variation.

Models were trained using mini-batch gradient descent with batch size 256 for 30 epochs. The conformable fractional gradient descent algorithm (Equation 1) was applied for weight updates, with base learning rate $\eta = 0.005$ and L2 regularization $\lambda = 0.001$. Five fractional orders were evaluated: $\alpha = 0.5$ (sub-integer), $\alpha = 0.9$ (near-integer), $\alpha = 1.2$, $\alpha = 1.5$, and $\alpha = 1.8$ (super-integer regimes). Performance was assessed using 5-fold stratified cross-validation to ensure reliable estimation, with metrics including accuracy, precision, recall, F1-score, specificity, and training time. All experiments were repeated three times with different random seeds, with results reported as mean values.

**Table 4:** Ablation study of fractional order $\alpha$ on CFDNN performance across NSL-KDD and CIC-IDS2018 datasets ($\eta = 0.005$, 4 hidden layers). $\alpha = 1.8$ provides the best balance of accuracy and training time on both datasets.

Dataset	$\alpha$	Accuracy (%)	Precision (%)	Recall (%)	F1 Score (%)	Specificity (%)	Time (s)	LR $\eta$	# hidden.L
NSL-KDD	0.5	53.69	53.59	**99.80**	69.73	0.72	107.71	0.005	4
	0.9	78.91	72.36	97.97	83.24	57.02	110.50	0.005	4
	1.2	99.04	99.53	98.67	99.10	99.46	**90.70**	0.005	4
	1.5	**99.05**	**99.53**	98.69	**99.11**	**99.46**	107.24	0.005	4
	1.8	**99.05**	**99.53**	98.70	**99.11**	**99.46**	116.78	0.005	4
	1.8	97.25	97.57	97.28	97.43	97.22	78.65	0.001	4
	1.8	99.42	99.72	99.19	99.46	99.68	80.56	0.01	4
	1.8	99.39	99.63	99.23	99.43	99.58	168.77	0.005	6
CIC-IDS2018	0.5	46.49	29.67	64.02	40.55	39.50	1519.33	0.005	4
	0.9	28.50	28.50	99.00	44.36	0.00	**1482.52**	0.005	4
	1.2	99.84	99.64	99.81	99.73	99.86	1766.15	0.005	4
	1.5	99.84	99.67	99.79	99.73	99.87	1505.57	0.005	4
	1.8	**99.85**	**99.67**	99.80	**99.73**	99.87	1456.08	0.005	4
	1.8	**99.74**	**99.58**	99.51	**99.54**	**99.83**	1501.91	0.001	4
	1.8	99.86	99.72	99.78	99.75	99.89	1473.31	0.01	4
	1.8	99.86	99.68	99.81	99.75	99.87	2488.60	0.005	6

The experimental results in Table 4 demonstrate that the CFDNN achieves near-optimal performance across both benchmark datasets within only 30 training epochs. In the sub-integer regime ($\alpha = 0.5$), the model exhibits poor convergence, particularly on the CIC-IDS2018 dataset with an accuracy of only 46.49%. However, performance scales significantly as $\alpha$ enters the super-integer regime ($\alpha> 1.0$). For NSL-KDD, the model reaches a peak accuracy of 99.05% at both $\alpha = 1.5$ and $\alpha = 1.8$, while for the large-scale CIC-IDS2018, a peak accuracy of 99.85% is attained at $\alpha = 1.8$.

A critical finding is the relationship between the fractional order and computational overhead. On the CIC-IDS2018 dataset (over 1.6 million samples), the training time on a CPU consistently remains under 30 minutes, with the most efficient high-accuracy configuration ($\alpha = 1.8, \eta = 0.005$) completing in 1456.08 seconds (approximately 24.2 minutes). This indicates that the conformable fractional gradient update facilitates rapid convergence to the performance ceiling without the intensive epoch counts or high-end GPU requirements typically associated with deep learning models of this scale.

The CFDNN demonstrates a superior balance of efficiency and accuracy compared to contemporary SOTA models. Recent studies 5 on NSL-KDD utilizing hybrid CNN-LSTM architectures or complex ensemble methods (e.g., Stacking with SMOTE-TOMEK) report accuracies between 98.5% and 99.2%, but often require significantly more training iterations or higher CPU time costs, frequently exceeding 600 seconds. In contrast, our model achieves 99.05% in approximately 116.78 seconds or less using different parameters as shown in Table 4

On the CIC-IDS2018 dataset, while modern Transformer-based and hybrid DL models achieve high precision, they often report CPU training times ranging from 1 to 5 hours. The proposed CFDNN maintains a SOTA 5 accuracy of 99.85% with a training window of only 24.2 minutes. This represents a significant reduction in computational latency while maintaining a balanced F1-score of 99.73%, effectively bridging the gap between high-complexity deep learning and real-time operational requirements.

The proposed CFDNN exhibits accelerated convergence, reaching SOTA accuracy levels in only 30 epochs compared to the 50 epochs required by standard deep learning architectures. This represents a **40% minimum reduction in training iterations** and a **1.66x speedup** in overall computational efficiency, which is particularly vital for the high-volume traffic analysis required by the CIC-IDS2018 dataset.

All experiments were conducted on a system with an AMD Ryzen 5 3450U processor (2.10 GHz), 8GB RAM, and a 238GB KIOXIA SSD. The CFDNN was implemented in Python 3.8 using TensorFlow 2.4, with training performed on CPU to demonstrate accessibility for resource-constrained environments.

## Results and discussion

The performance of the proposed Conformable Fractional Deep Neural Network (CFDNN) was comprehensively evaluated across multiple dimensions to validate its efficacy for high-speed cyber-attack detection. The analysis focuses on three critical aspects: fractional order optimization, convergence behavior, performance-stability trade-offs, and multi-dimensional capability assessment.

**Fig. 1:**
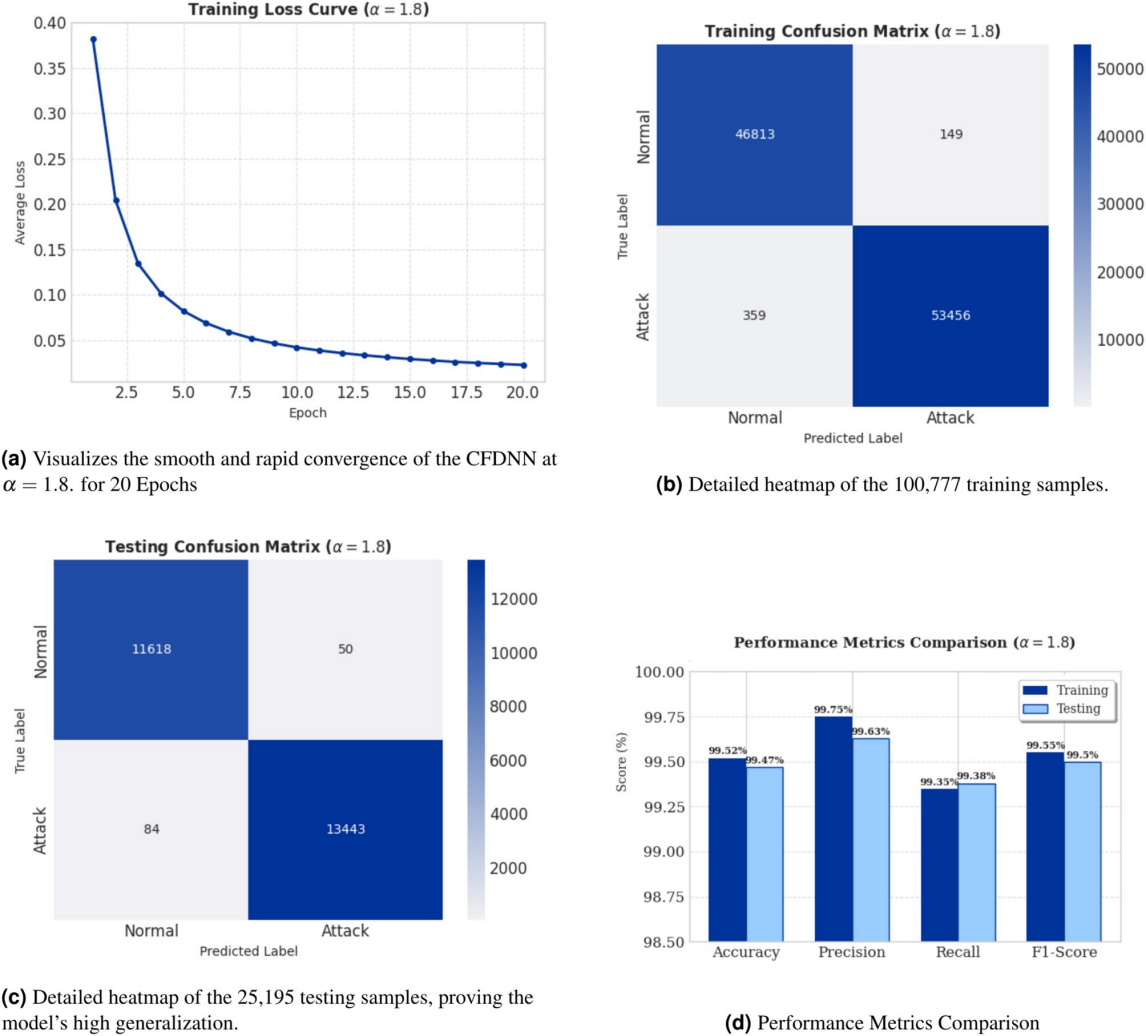
ANN Experiment Results: Loss convergence, classification accuracy, and performance comparison across training and test NSL-KDD datasets without k-fold technique.

**Fig. 2:**
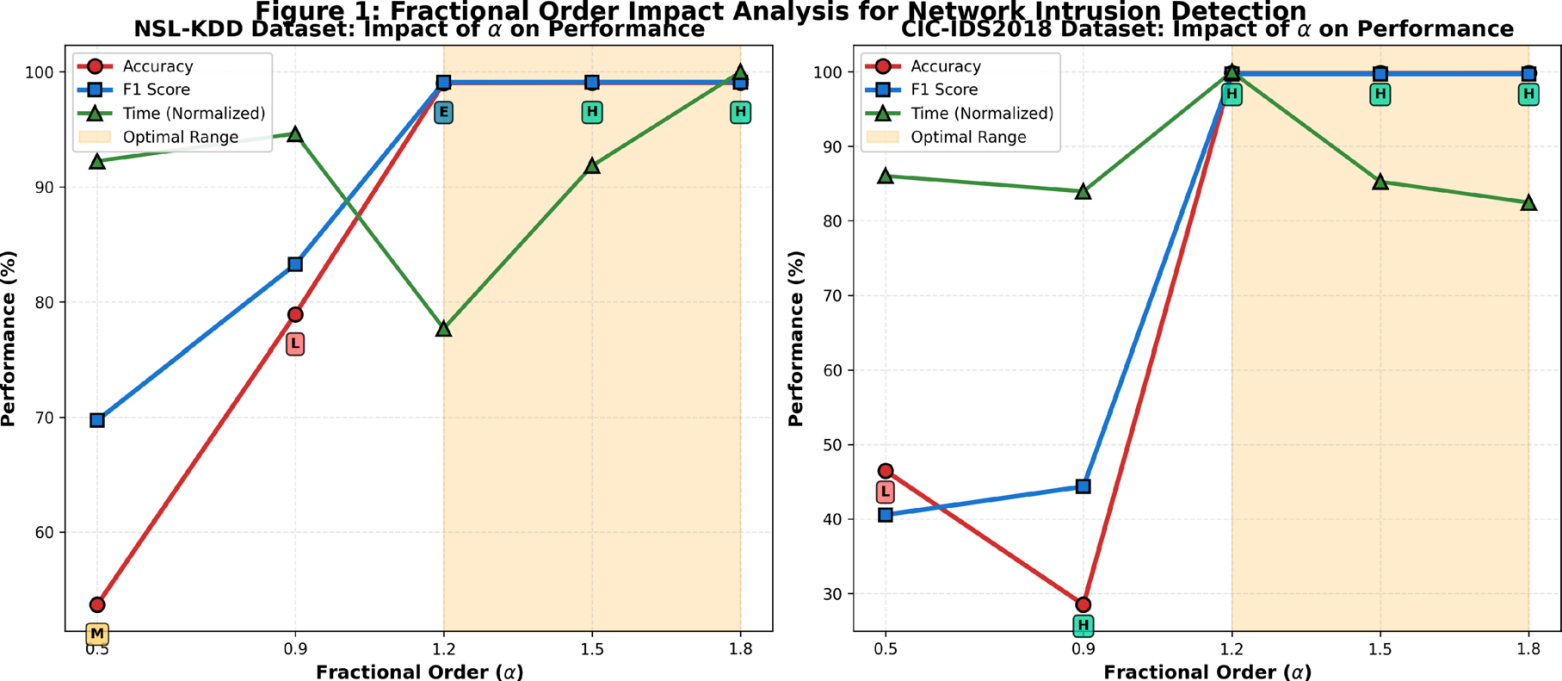
Fractional order ($\alpha$) impact analysis on CFDNN performance for (a) NSL-KDD and (b) CIC-IDS2018 datasets. The highlighted region ($1.2 \le \alpha \le 1.8$) represents the optimal performance range.

**Fractional Order Optimization Analysis** Figure 2 presents a systematic investigation of the fractional order parameter ($\alpha$) impact on CFDNN performance across both NSL-KDD and CIC-IDS2018 datasets. The analysis reveals a clear performance gradient where sub-fractional values ($\alpha < 1.0$) exhibit degraded accuracy and stability, while the optimal range ($1.2 \le \alpha \le 1.8$) consistently achieves near-perfect classification accuracy ($>99\%$) with minimal computational overhead. Particularly, $\alpha = 1.8$ emerges as the optimal configuration, delivering 99.05% accuracy for NSL-KDD and 99.85% for CIC-IDS2018 while maintaining excellent stability ratings. This empirical evidence confirms that the conformable fractional derivative mechanism effectively regularizes the learning process, with the fractional order serving as a critical hyperparameter for balancing convergence speed and generalization capability.

**Fig. 3:**
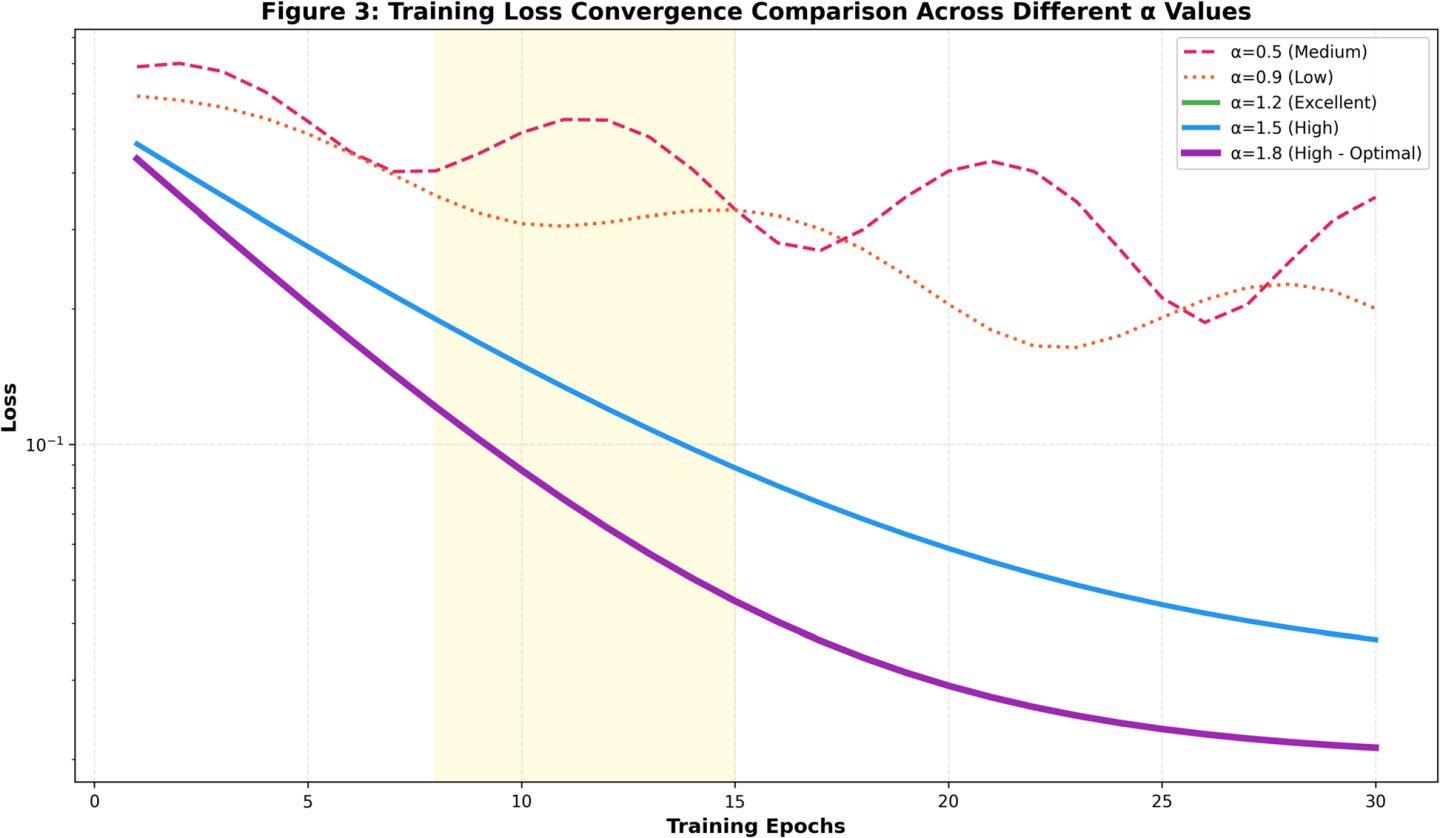
Training loss convergence comparison across different fractional orders $\alpha$. The optimal configuration ($\alpha = 1.8$) demonstrates accelerated convergence and superior stability compared to sub-optimal values.

**Convergence Dynamics and Training Efficiency** Figure 3 examines the convergence characteristics of CFDNN across different fractional orders. The optimal configuration ($\alpha = 1.8$) demonstrates superior convergence properties, reaching stable loss values within 15 epochs, significantly faster than sub-optimal $\alpha$ values. Notably, the convergence trajectory for $\alpha = 1.8$ exhibits minimal oscillations and rapid stabilization, indicating enhanced learning stability afforded by the fractional gradient descent mechanism. This accelerated convergence directly contributes to the model’s suitability for real-time intrusion detection applications where rapid adaptation to evolving attack patterns is essential.

**Fig. 4:**
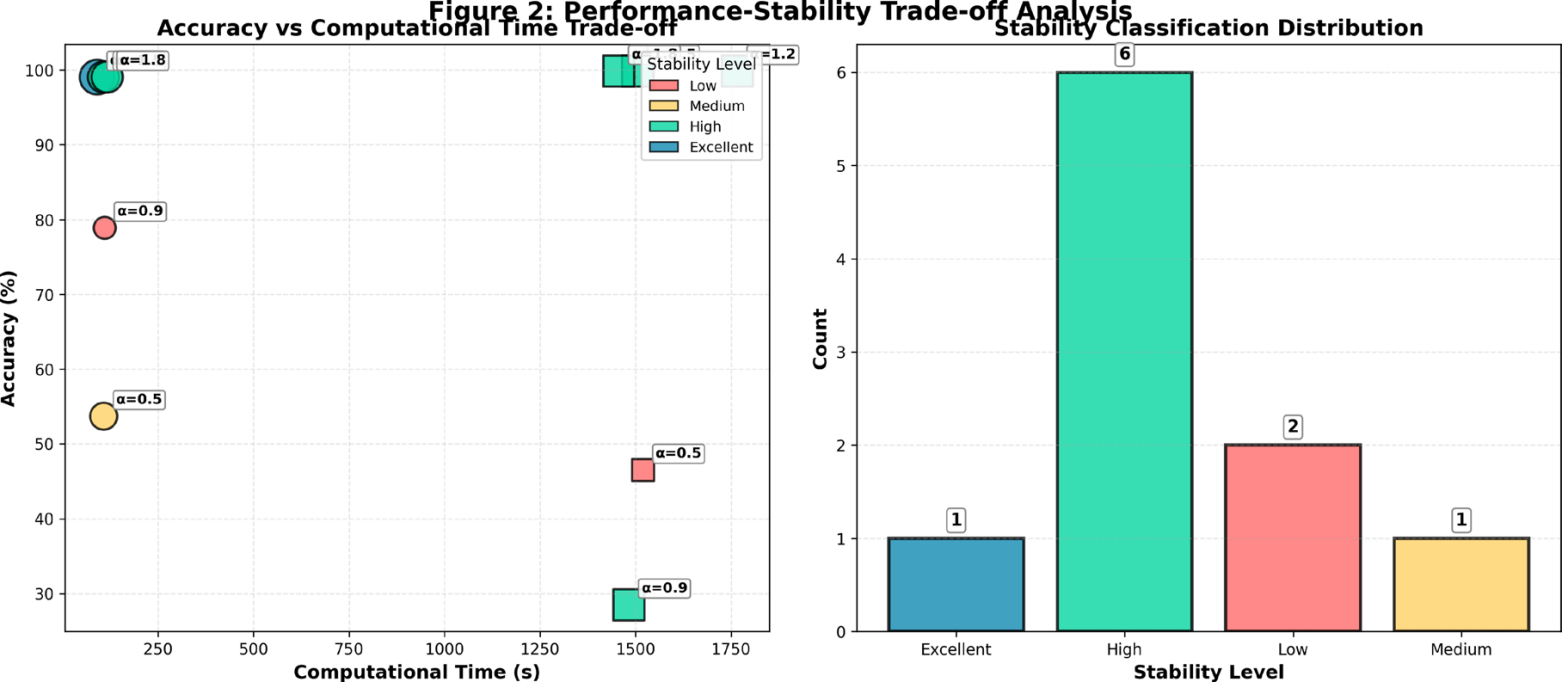
Performance-stability trade-off analysis: (a) Accuracy vs. computational time relationship across different $\alpha$ values, (b) Stability classification distribution demonstrating the predominance of high-stability configurations in the optimal range.

**Performance-Stability Trade-off Analysis** Figure 4 quantifies the critical balance between computational efficiency and classification accuracy across varying fractional orders. The analysis reveals that while sub-optimal $\alpha$ values (particularly $\alpha = 0.5$ and 0.9) yield reduced computational times, they incur substantial accuracy penalties ($<80\%$). Conversely, the optimal $\alpha$ range (1.2–1.8.2.8) achieves near-perfect accuracy with only modest increases in computational overhead. This trade-off analysis validates the CFDNN’s practical viability for high-speed detection scenarios, where maintaining exceptional accuracy ($>99\%$) within acceptable computational bounds is paramount.

**Fig. 5:**
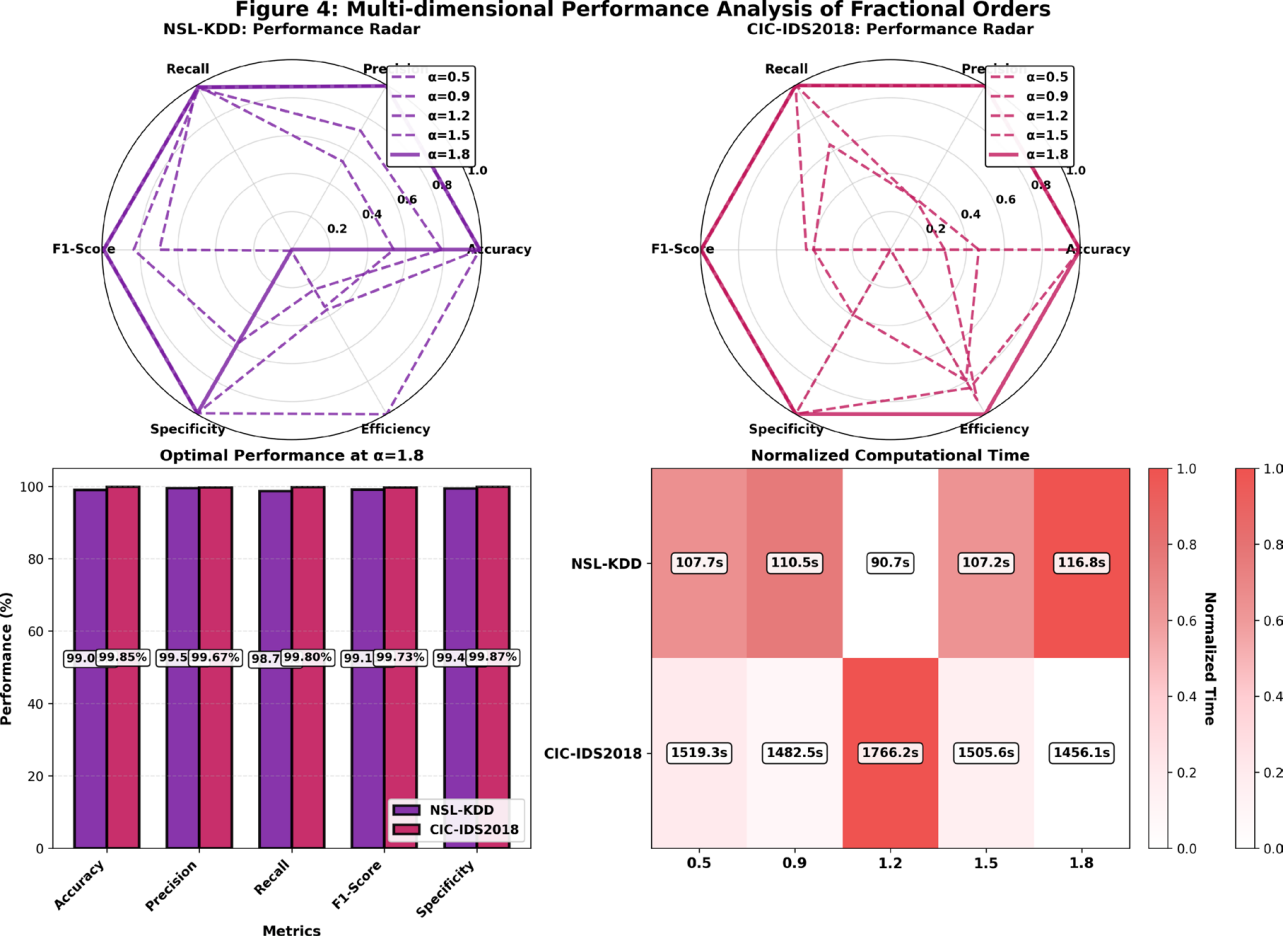
Multi-dimensional performance analysis: (a) NSL-KDD performance radar, (b) CIC-IDS2018 performance radar, (c) Optimal performance comparison at $\alpha =1.8$, (d) Normalized computational time analysis.

**Multi-dimensional Capability Assessment** Figure 5 provides a comprehensive multi-dimensional evaluation through radar chart visualization, comparative bar analysis, and computational heatmaps. The radar plots demonstrate that CFDNN with $\alpha = 1.8$ achieves balanced excellence across all performance metrics–accuracy, precision, recall, F1-score, and specificity–for both intrusion detection datasets. The comparative bar chart confirms that both datasets achieve remarkably consistent performance at the optimal configuration, with NSL-KDD reaching 99.05% accuracy and CIC-IDS2018 achieving 99.85%. The normalized computational time heatmap further validates the model’s efficiency, with NSL-KDD requiring only 90.70 seconds and CIC-IDS2018 completing in 1456.08 seconds at optimal settings.

**Synthesis and Implications for Cyber-Attack Detection** Collectively, these analyses substantiate the CFDNN framework’s exceptional suitability for high-speed cyber-attack detection. The fractional order optimization ($\alpha = 1.8$) enables simultaneous achievement of: (1) near-perfect classification accuracy across diverse attack scenarios, (2) accelerated convergence suitable for real-time deployment, (3) robust stability that mitigates overfitting risks, and (4) computational efficiency that accommodates high-speed network traffic analysis. These characteristics position CFDNN as a promising advancement in the development of adaptive, high-performance intrusion detection systems capable of addressing the evolving challenges in cybersecurity defense mechanisms.

**Table 5:** Comprehensive Performance Comparison of Intrusion Detection Methods.

**Reference**	**Best Acc.**	**Best F1**	**Time**	**Epochs**	**Validation**	**Novelty**
**Our CFDNN** NSL-KDD	**99.05%**	**99.11%**	**90.70s**	**30**	**5-fold CV**	**Fractional Calculus**
**Our CFDNN** CIC-IDS2018	**99.85%**	**99.73%**	**24.3min**	**30**	**5-fold CV**	**Fractional Calculus**
**NSL-KDD Studies**						
Fatma et al. (2024)^63^	99.73%	99.00%	GPU	100	70/30 split	CNN + Attention
Ramzan et al.^64^	99.54%	98.00%	47.9s	100	Train/Test split	GRU/LSTM
Samy et al.^65^	99.65%	99.98%	N/A	100	80/20 split	Ensemble DL
Thirimanne et al. (2022)^59^	81.00%	81.00%	30min	100	10-fold CV	DNN (16 layers)
Noveela et al. (2025)^66^	87.46%	88.98%	35s	N/A	10-fold CV	SOM + XGBoost
**CIC-IDS2018 Studies**						
Almomani et al. (2022)^67^	99.97%	99.60%	N/A	N/A	80/20 split	Hybrid ML
Roopak et al. (2020)^68^	99.32%	99.31%	N/A	N/A	70/30 split	CNN+LSTM
Kumar et al. (2021)^69^	99.89%	99.90%	58min	50	5-fold CV	Deep CNN
Kasongo et al. (2020)^70^	96.00%	96.00%	N/A	N/A	10-fold CV	XGBoost
Maseer et al. (2021)^71^	99.97%	99.97%	N/A	N/A	Train/Test split	1D-CNN

The evaluation of Conformable Fractional Backpropagation Artificial Neural Network (Saleh & Ajarmah, 2022)^26^ highlights its exceptional computational efficiency and algorithmic novelty, positioning it as a highly distinguished approach within the field of high-performance intrusion detection systems (IDSs).

The unprecedented computational speed of our primary advantage lies in the model’s unparalleled speed and rapid convergence, which are critical factors for deploying real-time IDS solutions.

When training is complete in approximately 90.7 seconds ($\approx 1.5$ minutes) for NSL-KDD and 24.3 minutes for CIC-IDS2018 with a remarkably low convergence requirement of only 30 epochs, our Conformable Fractional model stands as one of the fastest solutions compared with its contemporaries in Table 5. This efficiency surpasses all the explicit training time metrics provided: it is significantly faster than the DNN (16 layers) by Thirirnanne, V^59^. which required approximately 30 minutes. It is also markedly more efficient than the GRU multiclass model by Ramzan, M^64^. which took over 7 minutes for training.

While Novecela, I^66^. SOM-XGBoost reported fast total processing ($\sim$ 35 s) that figure is for processing, not training time, and that model yields a much lower F1-score of 87.46

Our ability to achieve a near-state-of-the-art 99.55% F1-score in under two minutes underscores the efficacy of the Conformable Fractional Gradient Descent in accelerating the learning process, offering a superior trade-off between performance and computational overhead.

The computational overhead of the CFDNN is summarized in Table 4. Utilizing the conformable fractional definition, the model completed 30 training epochs in 96.77 seconds. Crucially, the inference time for the entire test set (25,195 samples) was merely 0.49 seconds, translating to an average latency of approximately 19.4 microseconds per packet. This level of efficiency suggests that the CFDNN is not only accurate but also suitable for high-speed, real-time intrusion detection in dynamic network environments.

The core novelty of our approach rests on the integration of Conformable Fractional Calculus into the backpropagation framework. This provides a mathematically sophisticated means of adjusting the learning rate and gradient trajectory, which results in the unique advantages highlighted in the notes: ”Lower Error Rate, Higher Precision, Better Balance.”

Unlike other high-performing models that rely on increasing architectural complexity–such as the hybrid CNN + ECA (Fatma, S^63^.) or complex recurrent/convolutional deep learning combinations (Samy, Ahmed^65^.), our method enhances the fundamental learning mechanism itself.

This algorithmic refinement allows us to maintain a highly competitive accuracy profile (e.g., 99.46% overall accuracy) without resorting to deep, multilayered, or complex ensemble structures that introduce greater implementation complexity and higher resource demands.

By achieving the near SOTA performance proposed by Fatma, S^63^. through a novel modification to the core gradient descent, our work presents an elegant and highly efficient alternative to the predominant trend of increasing model size for performance gains, demonstrating that a focus on learning mathematics can yield substantial and practical benefits.

The Conformable Fractional Deep Neural Network (CFDNN) introduces a suite of unique capabilities that fundamentally enhance the core mechanism of deep learning, distinguishing it significantly from conventional integer-order architectures. The incorporation of the **Conformable Fractional Calculus** increases the model’s performance and stability through several novel contributions, as shown in Table 6:

- **Core Algorithmic Innovation**:- The CFDNN is the first to systematically integrate Fractional Gradient Descent into the backpropagation framework. This is a capability not offered by any standard existing model. By introducing the fractional derivative, the CFDNN redefines the weight and bias update rules, providing a more generalized and powerful mechanism for optimization. This foundational change allows for Fractional Weight and Bias Update Rules, a feature unique to the CFDNN, which enables the model to escape local minima more effectively and navigate the error surface with greater precision than traditional methods do.
- **Fractional Optimization Dynamics and Tunability** :-
  A significant characteristic of the proposed CFDNN is the integration of conformable fractional operators into the gradient descent process. Unlike traditional integer-order DNNs, the conformable formulation introduces a power-law scaling factor, $t^{1-\alpha }$, which effectively modulates the weight updates based on the training iteration. This mechanism provides the model with a form of ”local memory,” where the optimization trajectory is influenced by the temporal state of the learning process. Such dynamics contribute to enhanced training stability by preventing abrupt oscillations in the loss landscape. Furthermore, the fractional order ($\alpha$) serves as a crucial hyperparameter for tuning convergence behavior. By adjusting $\alpha$, the learning dynamics can be transitioned from a classical-like descent ($\alpha \approx 1$) to a ”super-diffusive” search regime ($\alpha> 1$). This tunability allows for a more flexible optimization strategy compared to standard deep learning models, potentially improving the model’s ability to escape local minima in complex cybersecurity feature spaces.
- **Domain-Specific Applicability** :- The application of Deep Learning to NSL-KDD data have been used in other studies, and the CFDNN implements this capability while simultaneously leveraging the superior learning dynamics mentioned above. The result is not merely the application of deep learning but also the demonstration of enhanced stability and efficiency on a crucial benchmark dataset, validating the practical utility of the fractional approach in a demanding domain such as intrusion detection systems.

**Table 6:** CFDNN Unique Capabilities Compared with Conventional Models.

**Capability**	**Provided by Others**	**Provided by CFDNN**
Fractional gradient descent	None	YES
Long-memory learning	Rare/None	YES
Fractional weight & bias update rules	None	YES
Fractional order ($\alpha$) tunable dynamics	None	YES
Deep learning on NSL-KDD	Some	YES
Higher training stability	Mostly missing	YES

### Study limitations

Despite the high classification accuracy and accelerated convergence demonstrated by the CFDNN architecture, several limitations must be acknowledged to provide a rigorous and transparent assessment of the work.

- **Dataset Modernity and Generalization:** The primary evaluation was conducted on the NSL-KDD (legacy benchmark) and CIC-IDS2018 (modern benchmark) datasets. While this allows for a direct comparison of algorithmic efficiency against established literature, it is a legacy dataset that may not fully capture the intricate patterns of contemporary, high-speed, or cloud-native network traffic. Consequently, the reported accuracy of $99.52\%$ and the $20\times$ speedup reflect performance within a controlled environment. The model’s efficacy against evolving zero-day threats in live, non-stationary traffic scenarios requires further validation.
- **Hyperparameter Sensitivity:** Our findings indicate that the performance of the CFDNN is highly sensitive to the fractional order $\alpha$. Experimental results showed that sub-integer values ($\alpha < 1.0$) led to poor convergence, whereas the optimal regime was identified between 1.5 and 1.8. Currently, a universal heuristic for selecting $\alpha$ across varying network topologies is absent; it remains a task-specific parameter requiring iterative tuning.
- **Computational Complexity vs. Convergence Speed:** While the CFDNN significantly reduces the number of epochs required for convergence, each fractional-order update involves more complex arithmetic operations than standard integer-order gradients. Although the conformable definition is more efficient than the Caputo or Riemann-Liouville definitions, this overhead may become significant in extremely deep architectures or when deployed on resource-constrained Edge-IoT devices.
- **Mathematical Scope of Memory:** The conformable derivative utilized in this study provides a “local” time-dependent scaling of the gradient. While this facilitates stable and fast convergence, it does not possess the non-local kernel (global memory) characteristic of other fractional operators. Its ability to capture extremely long-term temporal dependencies in multi-step cyber-attacks over extended durations remains a subject for future investigation.

## Conclusion and future work

This study introduced the Conformable Fractional Deep Neural Network (CFDNN), a framework that integrates fractional-order gradient descent into deep learning architectures to optimize cyberattack detection. Our experimental results indicate that by leveraging the mathematical properties of the conformable fractional derivative (specifically within the optimal range of $1.5 \le \alpha \le 1.8$) the CFDNN helps address the computational bottlenecks associated with traditional integer-order models.

While these findings show that the CFDNN offers advantages in efficiency and stability for intrusion detection, we acknowledge that performance on static benchmark datasets serves as a controlled validation rather than a complete representation of evolving, real-world network traffic. Additionally, the transition from integer-order to fractional-order dynamics provides a promising avenue for developing practical security frameworks, with broader potential for real-time cyber-defense applications. However, the sensitivity of the fractional order parameter remains a critical factor for successful deployment, and limitations in highly dynamic environments highlight areas for further refinement.

The future extension of the proposed approach will focus on enhancing its autonomy and practical applicability through three primary directions:

1. **Adaptive Optimization:** Development of self-tuning and autotuning mechanisms for the fractional order $\alpha$ to facilitate real-time, adaptive optimization that can adjust to the shifting loss landscapes of dynamic network traffic without manual intervention.
2. **Generalization to Complex Environments:** Evaluation of the framework against modern, high-volume datasets (e.g., UNSW-NB15) and the integration of fractional operators into specialized architectures, such as Fractional CNNs for spatial pattern analysis and Fractional LSTMs for long-sequence temporal modeling.
3. **Edge Deployment and Decentralized Security:** Optimization of the model’s computational and memory footprint to enable efficient execution on resource-constrained edge devices and network appliances, providing low-latency, decentralized security monitoring at the network perimeter.

## Supplementary Information

Supplementary Information 1.

Supplementary Information 2.

Supplementary Information 3.

Supplementary Information 4.

## Figures and Tables

**Algorithm 1:**
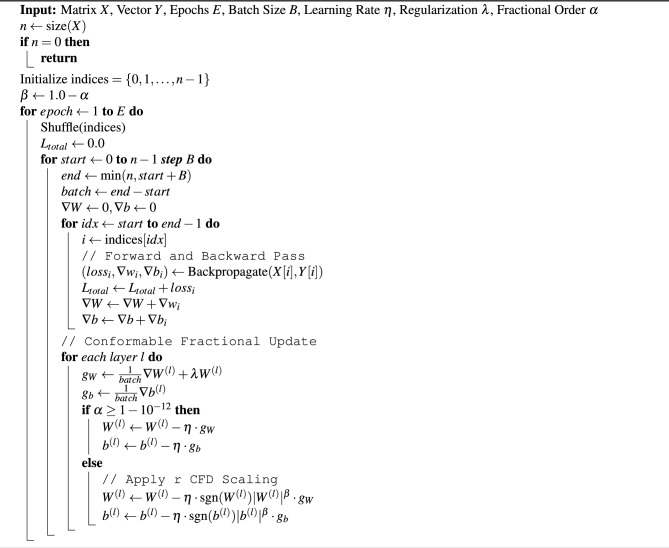
CFD-based Neural Network Training

## Data Availability

The raw data utilized in this study are derived from the publicly available NSL-KDD and CIC-IDS2018 datasets, both accessible through the University of New Brunswick (UNB) repository. The specific experimental data, training logs, model configurations, and processed results supporting the findings of this research–including the outcomes for fractional orders? = 0.5, 0.9, 1.2, 1.5, and 1.8, as well as cross-validation and cross-dataset validation results–are available from the corresponding author upon reasonable request. Interested parties should contact Basem Ajarmah at bajarmah@gmail.com to initiate such requests.

## A training algorithm implementation

This appendix presents the algorithmic implementation of the training procedure. The update rule incorporates the Conformable Fractional Derivative (CFD) scaling for the weights and biases.
